# Enhanced rigid-bond restraints

**DOI:** 10.1107/S0108767312014535

**Published:** 2012-05-01

**Authors:** Andrea Thorn, Birger Dittrich, George M. Sheldrick

**Affiliations:** a Department of Structural Chemistry, University of Göttingen, Tammannstrasse 4, D-37077 Göttingen, Germany

**Keywords:** rigid-bond test, refinement restraints, anisotropic displacement parameters

## Abstract

The rigid-bond model [Hirshfeld (1976 ▶). *Acta Cryst.* A**32**, 239–244] states that the mean-square displacements of two atoms are equal in the direction of the bond joining them. This criterion is widely used for verification (as intended by Hirshfeld) and also as a restraint in structure refinement as suggested by Rollett [*Crystallographic Computing* (1970 ▶), edited by F. R. Ahmed *et al.*, pp. 167–181. Copenhagen: Munksgaard]. By reformulating this condition, so that the relative motion of the two atoms is required to be perpendicular to the bond, the number of restraints that can be applied per anisotropic atom is increased from about one to about three. Application of this condition to 1,3-distances in addition to the 1,2-distances means that on average just over six restraints can be applied to the six anisotropic displacement parameters of each atom. This concept is tested against very high resolution data of a small peptide and employed as a restraint for protein refinement at more modest resolution (*e.g.* 1.7 Å).

## Abstract 2

An extension is proposed to the rigid-bond description of atomic thermal motion in crystals.

## 1. Introduction

The rigid-bond criterion (Hirshfeld, 1976 ▶) plays a key role in validating, understanding and restraining atomic displacement parameters (ADPs) in both small-molecule and macromolecular crystal structures. Its validity was established by Rosenfield *et al.* (1978 ▶), Dunitz, Maverick & Trueblood (1988 ▶), Dunitz, Schomaker & Trueblood (1988 ▶), Bürgi (1989 ▶) and many others. It plays an important part in the validation of crystal structures using *PLATON* (Spek, 2009 ▶) and the IUCr CheckCIF suite. Rollett (1970 ▶) was probably the first to apply rigid-bond *restraints* in the least-squares refinement of crystal structures by means of additional observational equations. This rigid-bond restraint [DELU in *SHELXL* (Sheldrick, 2008 ▶) or RBON in *REFMAC* (Murshudov *et al.*, 2011 ▶)] is useful both for the treatment of positional disorder in small-molecule structures and for enabling anisotropic refinement of macromolecules at relatively good resolution.

The rigid-bond criterion states that the mean-square displacement amplitudes of bonded atoms are equal in the direction of the bond joining them, and it is often applied to 1,3-distances (involving two atoms that are both bonded to a common atom) as well. When applied as a restraint, the standard deviation is usually set to a value in the range 0.01 to 0.001 Å^2^, a value that is consistent with the deviations observed in structures in which an anisotropic refinement is possible without restraints. However, it only provides about one restraint per atom, which is not enough to compensate for the six degrees of freedom per atom associated with the anisotropic displacement parameters *U*_*ij*_. In practice it needs to be supplemented by other ADP restraints. By also applying it to 1,3-bonded atoms, the number of restraints can be increased to an average of about two per atom. It is usually necessary to apply further restraints, for example that the atom is approximately isotropic (ISOR in *SHELXL*) and that the *U*_*ij*_ values are equal to the corresponding *U*_*ij*_ values of spatially close atoms (the similarity restraint SIMU). Since these restraints are much less justified by theory and experimental evidence, they are given large estimated standard deviations, but at least they add about six restraints (SIMU) or five (ISOR) per atom. The similarity restraints enable a stable refinement of severely overlapping disorder components, but in practice they are rather approximate descriptions of the real atomic motion. Either these restraints are made too tight, and the *R* factors are high, or (typically for macromolecular refinements) the restraints are too slack and the gap between the *R* values of the working set and the test set (the free *R*; Brünger, 1992 ▶) becomes large, indicating over-refinement.

In this paper a simple extension of the rigid-bond concept will be discussed.

## 2. The enhanced rigid-bond criterion

**Figure 1:**
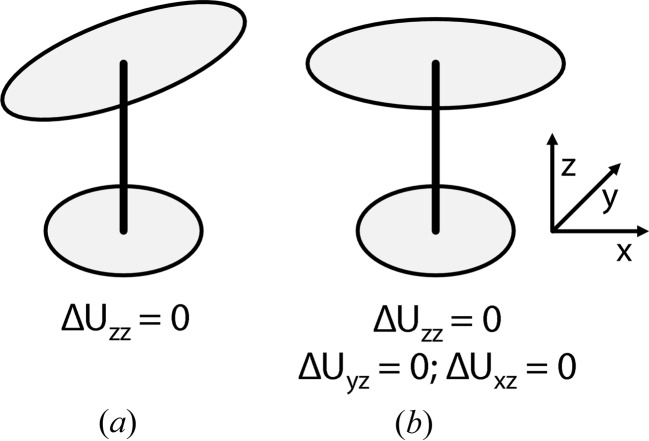
The conventional rigid-bond criterion is satisfied by both (*a*) and (*b*), but only (*b*) fulfils the enhanced rigid-bond restraint.

Fig. 1 ▶(*a*) illustrates the thermal ellipsoids for a bonded pair of atoms that would satisfy the usual rigid-bond criterion; the mean-square displacements of the two atoms along the bond are equal. However, if the bond is really rigid, the relative motion of the two atoms should be perpendicular to the bond, as in Fig. 1 ▶(*b*). To express this additional information in the form of a restraint, we need to transform the *U*_*ij*_ to a local orthogonal axis system in which the *Z* axis is along the bond, *Y* is an arbitrary direction at right angles to *Z*, and *X* is at right angles to both *Y* and *Z*. Restraints are then applied to the *differences* of the transformed components *U*_*ZZ*_, *U*_*XZ*_ and *U*_*YZ*_: 
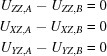
but not to the differences of the other three components (if all six differences were restrained in this way, a SIMU similarity restraint would be produced). In this way the number of restraints per atom is multiplied by three, and if such restraints are also applied to the 1,3-distances, there are about six reliable restraints for the six *U*_*ij*_ components of each atom, and it may not even be necessary to apply other ADP restraints!

### 2.1. Verification at very high resolution

In order to assess the suitability of the enhanced rigid-bond criterion for macromolecular refinement, a high-quality synchrotron data set of a cyclic hexapeptide measured to a resolution of 0.382 Å at a temperature of 100 K was employed. A standard *SHELXL* IAM (independent-atom model) refinement was performed both for the full data and for data truncated to 0.84 Å, and compared with the published results (Dittrich *et al.*, 2002 ▶) from multipole refinement with the program *XD* (Koritsánszky *et al.*, 2003 ▶) that used the full 0.382 Å data.

For the conventional rigid-bond criterion, the numbers in the first row of Table 1 ▶ should be close to zero; for the extension proposed here, the first two rows should be close to zero, and for the SIMU restraint in *SHELXL* all four rows should have zero values. Although the multipole refinement should give more accurate ADPs because it takes the bonding electrons into account, the differences in the transformed *U*_*ij*_ components are extremely similar for the full data for the two approaches, as shown in Table 1 ▶. However, the agreement with all the *U*_*ij*_ criteria (*i.e.* the differences from zero) was significantly worse when the data were truncated to a ‘normal’ resolution of 0.84 Å, presumably because the ADPs are compensating for the inadequately modelled bonding electron density, which primarily affects the low-angle data. It can be seen that the additional conditions proposed here for the rigid-bond restraint (second row) are less exactly fulfilled than the conventional rigid-bond restraint itself, but fit better than the conditions for the SIMU similarity restraint. It can also be seen that the conditions apply to 1,3-distances but less precisely than for 1,2-distances. When these conditions are applied as restraints, their estimated standard deviations should reflect the trends shown in Table 1 ▶. Since 1,4-distances could be affected by torsional motion, an appreciably larger estimated standard deviation would be required if such restraints were also applied to them.

### 2.2. Application as a refinement restraint at modest resolution

The data-to-parameter ratio only permits free anisotropic refinement of macromolecules at resolutions similar to those encountered for small molecules, which are almost always refined anisotropically (0.84 Å or better). For resolutions in the intermediate range (0.8 Å to about 1.6 Å) a restrained anisotropic refinement is often performed (in the case of *SHELXL*, with the DELU, SIMU and ISOR restraints), and for lower resolutions a hybrid model based on TLS (Schomaker & Trueblood, 1968 ▶; Holbrook & Kim, 1984 ▶; Howlin *et al.*, 1989 ▶) plus additional (possibly restrained) isotropic displacement parameters (Winn *et al.*, 2001 ▶) is popular. The enhanced rigid-bond restraint (with the *SHELX* keyword RIGU) should be suitable for the intermediate range and might enable restrained anisotropic refinement to be extended to lower resolution.

In order to apply the new (RIGU) restraints, it was first necessary to find suitable estimated standard deviations. Two models were tested, based on experience with other ADP restraints (Thorn, 2011 ▶). In the first, the standard deviations were set to [(*p* ^2^ + *U*_eq,*A*_ + *U*_eq,*B*_)]^1/2^σ/*p* and in the second to [(*p* ^2^ + *U*_eq,*A*_ + *U*_eq,*B*_)]^1/2^σ*d*/*p*, where *A* and *B* are the two atoms, *U*_eq_ is the equivalent isotropic displacement parameter (Watkin, 2000 ▶), and *d* is the distance between atoms *A* and *B*. σ and *p* are user-supplied parameters. Taking the *U*_eq_ values into account in this way slackens the restraints for large displacement parameters that may well reflect partial disorder as well as thermal motion. Experiments showed that the second formula with σ = 0.004 Å^2^ and *p* = 0.5 gave good results, though the actual values of the parameters were not very critical, and these values were used for the tests reported here. The incorporation of the distance *d* has the effect of weighting down the 1,3-restraints relative to the 1,2-restraints.

A set of eight well refined high-resolution structures with data to resolutions in the range 0.7 to 1.2 Å and between 55 and 331 amino-acid residues in the asymmetric unit was used to test the enhanced rigid-bond restraint (PDB codes: 1b0y, 1lu0, 1ok0, 1rqw, 1us0, 2cm5, 2fdn and 2vb1). In all cases the minor components of disordered residues were removed and the water molecules were refined isotropically, so the *R* factors are a little higher than the published values. Before refinement the structures were ‘shaken’ by applying random shifts (with a mean of 0.5 Å) to each atom to remove memory effects and refined to convergence. Good convergence was still obtained except when the σ values were too large (*i.e.* the ADP restraints were too weak).

**Figure 2:**
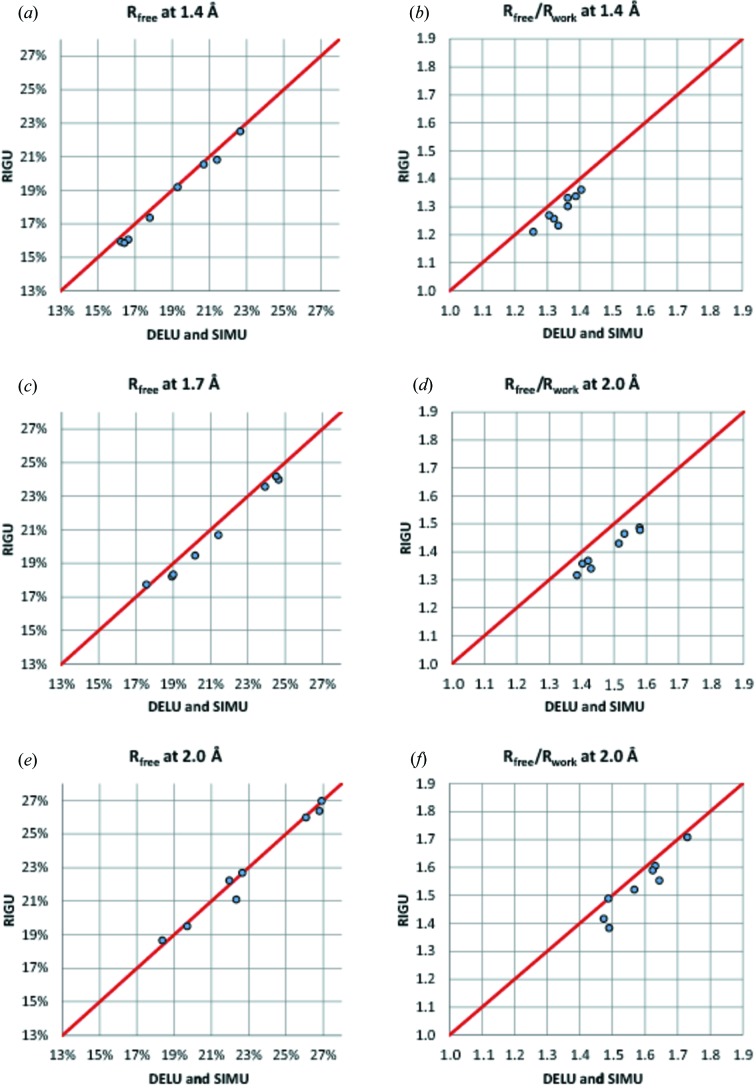
The free *R* factors (*a*), (*c*), (*e*) and the ratios *R*_free_/*R*_work_ (*b*), (*d*), (*f*) for data truncated to 1.4 Å (*a*), (*b*), 1.7 Å (*c*), (*d*) and 2.0 Å (*e*), (*f*) for the new (RIGU) restraints compared to the standard *SHELXL* DELU and SIMU restraints with their default settings.

**Figure 3:**
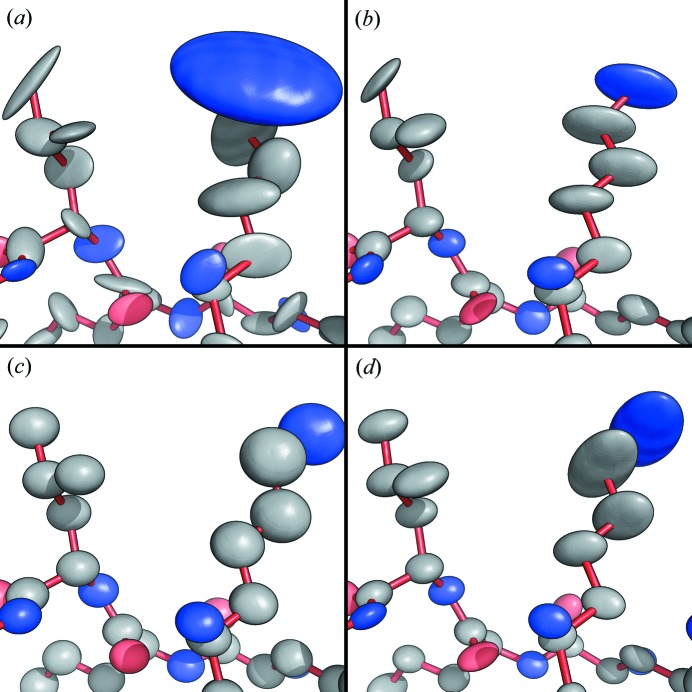
Side-chain detail from the human aldose reductase structure (Howard *et al.*, 2004 ▶; PDB code 1us0) for data truncated to 1.7 Å showing 50% probability ellipsoids after refinement with (*a*) no restraints, (*b*) standard *SHELXL* DELU and SIMU restraints, (*c*) TLS and additional isotropic *B* values using *REFMAC*, and (*d*) the new RIGU restraints.

The tests summarized in Table 2 ▶ and Fig. 2 ▶ for the eight test structures show that both the enhanced rigid-bond restraints (RIGU) and the standard combination of SIMU and DELU restraints give significantly lower free *R* factors than unrestrained isotropic refinement at all three resolutions tested (1.4, 1.7 and 2.0 Å). The free *R* factors (Brünger, 1992 ▶) for the RIGU refinements are slightly lower than those for SIMU + DELU, but the ratio of *R*_free_ to *R*_work_ (Tickle *et al.*, 1998 ▶) is significantly better for RIGU, indicating that it suffers less from over-refinement. Fig. 3 ▶ compares the thermal ellipsoids for the different refinements. Without restraints, the chaotic ellipsoids bear little relation to physical reality. The DELU + SIMU refinement is already a considerable improvement, but the finer details of the RIGU refinement are more realistic, with the relative motion of the atoms being more perpendicular to the bonds (compare Fig. 1 ▶). It should be noted that the e.s.d.’s for the SIMU restraints have to be set higher than for RIGU to obtain acceptable free *R* values. The TLS refinement was performed with *REFMAC* version 5.6.0.117 (Murshudov *et al.*, 2011 ▶) instead of *SHELXL* and so is not directly comparable. However, as can be seen in Fig. 3 ▶(*c*), there is a general tendency for the atoms at the periphery of the molecule to appear as spheres rather than ellipsoids, This is characteristic of the TLS plus additional iso­tropic ADP model, because the ADPs of these atoms are dominated by the additional isotropic contributions.

## 3. Conclusions

The enhanced rigid-bond model provides a realistic description of the molecular motion, and is likely to find application both for small-molecule structure verification and as a restraint in the anisotropic refinement of both small molecules and macromolecules when disorder is present or the effective data-to-parameter ratio does not permit unrestrained anisotropic refinement. One such application would be for powder rather than single-crystal data of small molecules. The more realistic description of the atomic thermal motion means, however, that the ADPs will not be able to compensate so well for unmodelled disorder and other problems. There is good evidence (MacArthur & Thornton, 1999 ▶; Lang *et al.*, 2010 ▶) that protein structures possess many more alternative low-occupancy side-chain conformations than are usually modelled. The extra (unrestrained) isotropic displacement parameters in the widely used TLS models for ADP refinement of proteins are, in contrast to the rigid-bond restraints, well able to compensate for the resulting unequal occupancies of the atoms involved. This helps to explain why the hybrid TLS model is so effective. On the other hand, the enhanced rigid-bond model should be better able to expose defects in the model, especially those involving previously undetected alternative conformations or chemical inhomogeneity.

## Figures and Tables

**Table 1:** Root-mean-square (r.m.s.) differences in transformed displacement parameters for 1,2- and 1,3-bonded atoms for *SHELXL* IAM and *XD* multipole refinements of a hexapeptide

	IAM 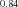 1,2	IAM 1,2	*XD* 1,2	IAM 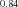 1,3	IAM 1,3	*XD* 1,3
r.m.s. Δ*U*_*ZZ*_ (Å^2^)	0.00165	0.00034	0.00031	0.00188	0.00074	0.00076
r.m.s. Δ*U*_*XZ*_ and Δ*U*_*YZ*_ (Å^2^)	0.00258	0.00180	0.00183	0.00301	0.00242	0.00240
r.m.s. Δ*U*_*XY*_ (Å^2^)	0.00406	0.00304	0.00304	0.00389	0.00386	0.00393
r.m.s. Δ*U*_*XX*_ and Δ*U*_*YY*_ (Å^2^)	0.00665	0.00605	0.00600	0.00788	0.00761	0.00767

**Table 2:** Average values of *R*_free_ (%) (Brünger, 1992 ▶) and *R*_free_/*R*_work_ (Tickle *et al.*, 1998 ▶) for eight test protein structures with different ADP restraints and data truncated to different resolutions

	1.4 Å	1.7 Å	2.0 Å
Unrestrained isotropic	20.9/1.25	22.2/1.38	24.3/1.59
RIGU	18.5/1.29	20.8/1.41	22.9/1.53
DELU + SIMU	18.9/1.34	21.3/1.48	23.1/1.58
